# Effect of individual food preferences on oscillatory brain activity

**DOI:** 10.1002/brb3.1262

**Published:** 2019-04-04

**Authors:** Nachie Tashiro, Hisato Sugata, Takashi Ikeda, Kojiro Matsushita, Masayuki Hara, Kenji Kawakami, Keisuke Kawakami, Minoru Fujiki

**Affiliations:** ^1^ Department of Neurosurgery, Graduate School of Medicine Oita University Oita Japan; ^2^ Faculty of Welfare and Health Science Oita University Oita Japan; ^3^ Research Center for Child Mental Development Kanazawa University Kanazawa Japan; ^4^ Department of Mechanical Engineering Gifu University Gifu Japan; ^5^ Graduate School of Science and Engineering Saitama University Saitama Japan

**Keywords:** EEG, food preferences, oscillatory brain activity, Pre‐oral (anticipatory) phase, swallowing

## Abstract

**Objectives:**

During the anticipatory stage of swallowing, sensory stimuli related to food play an important role in the behavioral and neurophysiological aspects of swallowing. However, few studies have focused on the relationship between food preferences and oscillatory brain activity during the anticipatory stage of swallowing. Therefore, to clarify the effect of individual food preferences on oscillatory brain activity, we investigated the relationship between food preferences and oscillatory brain activity during the observation of food images.

**Methods:**

Here we examined this relationship using visual food stimuli and electroencephalography (EEG). Nineteen healthy participants were presented 150 images of food in a random order and asked to rate their subjective preference for that food on a 4‐point scale ranging from 1 (don't want to eat) to 4 (want to eat). Oscillation analysis was performed using a Hilbert transformation for bandpass‐filtered EEG signals.

**Results:**

The results showed that the oscillatory beta band power on C3 significantly decreased in response to favorite foods compared to disliked food.

**Conclusion:**

This result suggests that food preferences may impact oscillatory brain activity related to swallowing during the anticipatory stage of swallowing. This finding may lead to the development of new swallowing rehabilitation techniques for patients with dysphagia by applying food preferences to modulate oscillatory brain activity.

## 1 INTRODUCTION

Eating is a basic human need as well as a source of pleasure for several people. Patients with dysphagia, which is characterized by swallowing difficulties, suffer a significant decrease in quality of life (QOL). Dysphagia occurs in aspiration pneumonia, a type of lung infection caused by the aspiration of solids and liquids into the lungs, and may cause death. In Japan, pneumonia is the third leading cause of death as per the Vital Statistics, Ministry of Health, Labor and Welfare, 2016. Thus, it is important to clarify the swallowing function to improve QOL and reduce mortality in patients with dysphagia.

Current studies related to swallowing have adopted a variety of perspectives (Ertekin & Aydogdu, 2003; Jestrović, Coyle, & Sejdić, 2015; Kern, Jaradeh, Arndorfer, & Shaker, 2001; Loret, 2015; Michou & Hamdy, 2009; Yang, Ang, Wang, Phua, & Guan, 2016). For example, Maeda et al. (2004) reported that a drink‐related visual stimulus facilitated the initiation of voluntary swallowing. In addition, Yoshikawa, Tanaka, Ishii, Yamano, and Watanabe (2016) reported that a series of visual food stimuli had a significant impact on resting state oscillatory brain activity in the beta band in the insula, dorsolateral prefrontal cortex (DLPFC), orbitofrontal cortex (OFC), and frontal pole. These results suggest that food stimulus may modulate behavioral and neurophysiological aspects related to swallowing. In other words, food visual stimulus may affect the oscillatory brain activity related to the anticipatory stage of swallowing. In addition, other studies have reported the importance of sensory stimuli, including smell, taste, and texture, during the anticipatory stage of swallowing (Mistry, Rothwell, Thompson, & Hamdy, 2006; Pelletier & Dhanaraj, 2006; Steele & Miller, 2010). Taken together, these studies suggest that sensory stimuli related to food play an important role in the swallowing function, including behavioral and neurophysiological aspects (Jestrović et al., 2015; Maeda et al., 2004; Michou & Hamdy, 2009; Yoshikawa et al., 2016).

Conversely, many studies focusing on the oscillatory brain activity have revealed the neural mechanisms associated with various types of motor and cognitive processes by determining the attenuation of cerebral oscillatory power [known as event‐related desynchronization (ERD)] (Palva & Palva, 2007; Sugata et al., 2017; Ward, 2003). Particularly, some studies have investigated the neural profiles for low‐frequency components such as alpha and beta bands (Neuper, Wörtz, & Pfurtscheller, 2006; Rektor, Sochůrková & Bocková, 2006; Klostermann et al., 2007), and have shown that these frequencies have a functional diversity in cortico‐basal networks that are simultaneously activated during sensorimotor processing (Klostermann et al., 2007). For example, alpha oscillation substantially influences perception, behavior, and neuronal processing (Mathewson et al., 2011), and beta oscillation reflects the maintenance of the current sensorimotor or cognitive state (Lalo et al., 2007; Meule, Kübler, & Blechert, 2013). In other words, the alpha band is related to sensory processing, and the beta band is associated with movement and somatosensory functions (Neuper et al., 2006; Rektor et al., 2006).

Given that sensory stimuli related to food affect neurophysiological as well as behavioral aspects of swallowing, it may be possible that individual food preferences affect oscillatory brain activity. However, few studies have focused on the relationship between food preferences and oscillatory brain activity during the anticipatory stage of swallowing. If the observation of favorite food could modulate oscillatory brain activity related to movement preparation, such as alpha and beta bands, and facilitate swallowing function, this may lead to the establishment of a new evidence that rehabilitation, taking into account individual food preferences, is effective for patients with dysphagia. Here, we hypothesized that food preferences affect oscillatory brain activity in alpha and beta bands during the anticipatory stage of swallowing. To test this hypothesis, we examined the relationship between food preferences and ERDs in alpha and beta bands activity related to the anticipatory stage of swallowing using food visual stimuli and electroencephalography (EEG).

## 2 MATERIALS AND METHODS

### 2.1 Participants

A total of 19 healthy volunteers participated in this study (seven males and 12 females; mean age 24 ± 4.95 years). All participants were strongly right‐handed (as assessed by the Edinburgh Handedness Inventory; Oldfield 1971, score 97.6 ± 5.38), had normal or corrected‐to‐normal vision, and no history of neurological or psychiatric disorders. Also, the participants did not take any medications on the day of EEG recording.

The experiments were conducted according to the principles of the Declaration of Helsinki. We explained the purpose and possible consequences of this study to all participants and obtained their informed consent before commencing the experiments. This study was approved by the Ethical Review Boards of the Oita University Faculty of Medicine (approval number 1,258).

### 2.2 Experimental design

All experiments took place between 10 a.m. and 12 p.m. Participants were asked to refrain from eating for at least 3 hr before the study; therefore, participants either ate breakfast between 7 a.m. and 9 a.m. or did not eat breakfast.

Visual stimuli were selected from a food picture database featuring images with simple compositions for experimental research (Blecher, 2014; Meule et al., 2013) (also see www.food-pics.sbg.ac.at). To avoid bias toward individual food preferences, 75 high‐calorie (HC) and 75 low‐calorie (LC) food images were selected from the database. Previous studies have reported different brain activity during observation of high‐calorie and low‐calorie foods (Killgore et al., 2003; Toepel, Knebel, Hudry, Coutre, & Murray, 2009). HC food images included both sweet and savory foods. LC food images included vegetables, fruits, and salad. HC and LC food images did not differ in color (RGB), brightness, spatial frequencies and contrast (*p* < 0.05), and visual complexity (edge detection: *p* < 0.05). All images had the same resolution and color depth (600 × 450 pixels, 96 dpi, 24 bpp) and were homogenous with regard to background color and camera distance. Stimuli were presented at a 50 cm visual distance and a visual angle of 11.4 × 5.7 degrees with a refresh rate of 59 Hz.

**Figure 1:**
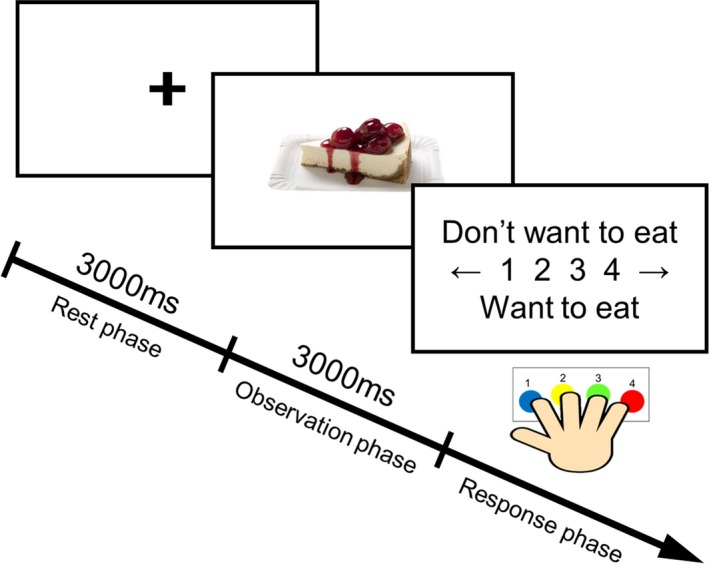
Experimental paradigm. Each epoch began with a 3,000 ms resting phase and visual presentation of a black fixation cross. A food picture was then presented for 3,000 ms. Afterwards, participants rated their subjective level of preferences for the food in the picture on a 4‐point scale from 1 (don't want to eat) to 4 (want to eat) by pressing buttons

The experimental paradigm is shown in Figure 1. An epoch started with 3,000 ms in the resting phase and visual presentation of a black fixation cross (rest phase). The food image was then presented for 3,000 ms (observation phase). After that, the participant was asked to rate their subjective level of preference for that food by pressing a button corresponding to a 4‐point response scale from 1 (don't want to eat) to 4 (want to eat) (response phase).

### 2.3 EEG measurements

EEG was recorded using active electrodes (Polymate Mini AP108, Miyuki‐giken, Japan). Eight electrodes (F3, Fz, F4, C3, Cz, C4, O1, and O2) were placed according to the international 10/20‐electrode system, and the electrode impedance did not exceed 20 kΩ. EEG electrodes were composed of sintered Ag/AgCl material. The ground electrode was located on the forehead and the reference was mounted on the left earlobe.

EEG data were sampled at a rate of 500 Hz with an online band‐pass filter at 1–100 Hz. Notch filtering was performed to eliminate AC line noise. To reduce noise due to muscle activity and eye movement, we instructed the participants to avoid unnecessary body movements and to focus on the center of the display without moving their eyes. After data acquisition, muscle activity‐contaminated trials were manually rejected during offline analysis. Furthermore, in order to confirm a signal to noise ratio (SNR), we calculated SNR using the individual averaged waveform. The background noise signal was defined from 1,000 to 0 ms, and target signal was defined from 0 to 1,000 ms. The average SNR was 11.0 ± 4.2 dB.

### 2.4 Analysis

All of the trials for each participant were divided into two groups: favorite food trials (FF) and disliked food trials (DF). Trials in which the participant pressed buttons 1/2 or buttons 3/4 were defined as DF and FF, respectively, because the number of presses for each of the four buttons was different, the number of trials was realigned using the following procedure. First, the smaller number of trials between 1 and 4, and between 2 and 3, was examined. Second, the number of button presses was realigned between 1 and 4 button presses and between 2 and 3 button presses using a smaller number of trials. Third, presses of buttons 1 or 2 (DF) and buttons 3 or 4 (FF) were concatenated. Lastly, the number of DF and FF trials became equivalent for each participant, and these data were used in the following analyses.

Time frequency analysis was applied to determine oscillatory brain activity during observation of food images for each participant using the Fieldtrip toolbox (www.fieldtriptoolbox.org). The recorded EEG signals were time‐locked at the onset of food image presentation (0 ms). The EEG signals of each electrode were bandpass‐filtered by frequency bands of 2 Hz width with 50% overlap. In accordance with a previous study (Yanagisawa et al., 2011), a Hilbert transformation was applied to the filtered signals to obtain complex‐valued analytic signals. The temporal power spectral density of each frequency band was calculated from the square of the absolute value of the complex‐valued signals. The extracted temporal powers were normalized by subtracting the mean of the baseline power (−200 to 0 ms from onset of food images). Next, we extracted the power of the alpha (8–13 Hz) and beta (13–25 Hz) bands to examine the temporal power change in each frequency band during observation of food images. We then compared the temporal power change in alpha and beta bands between FF and DF trials using the Student's *t* test at each time point. To control multiple comparisons, statistical *p* value was corrected by false discovery rate (FDR) over all time points (Benjamini & Hochberg, 1995). Based on the previous study that used FDR correction with q‐thresholds as high as 0.2 (Genovese, Lazar, & Nichols, 2002), we employed statistical testing with an FDR correction procedure at a *q*‐value of 0.15. This means that the statistically significant level of *p* value is 0.015. In addition, to examine whether there are asymmetries on oscillatory brain activity between left and right hemispheres, we compared the oscillatory brain activities between left and right EEG channels at 0–500 ms after the presentation of food stimulus using the Student's *t* test. Furthermore, to examine whether food preferences affected response time, we compared the response time between FF and DF trials. Lastly, we performed correlation analysis between oscillatory brain activity and response time using the Pearson's test to clarify the relationship between oscillatory brain activities and behavioral results.

## 3 RESULTS

**Figure 2:**
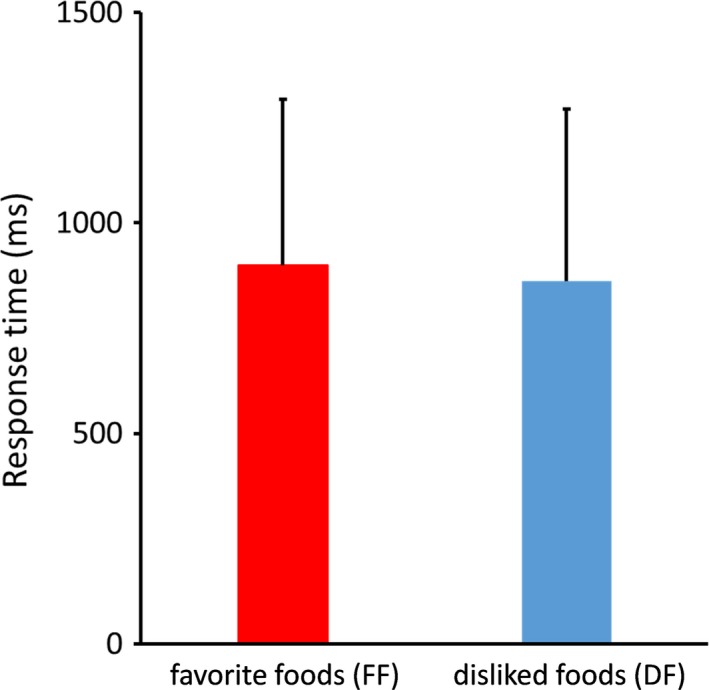
Response time during the response phase. No significant differences were observed between FF and DF

**Figure 3:**
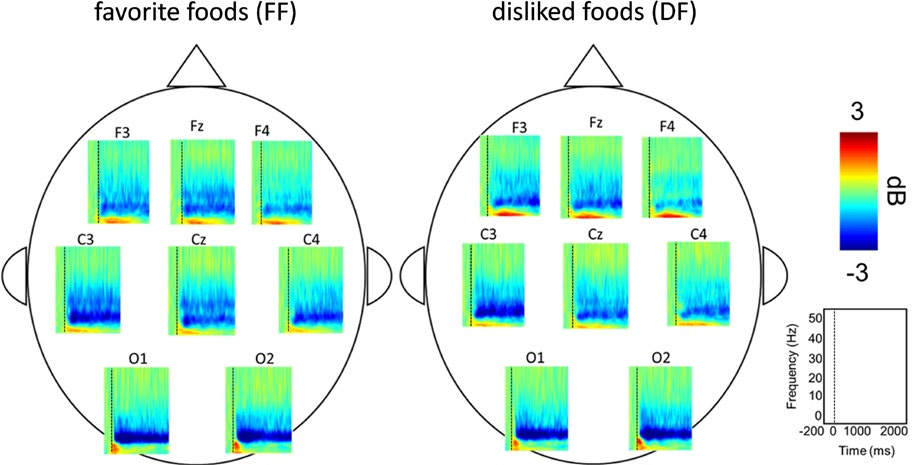
Grand averaged time frequency spectrograms. Robust ERDs were observed in the alpha and beta bands after presentation of food images

**Figure 4:**
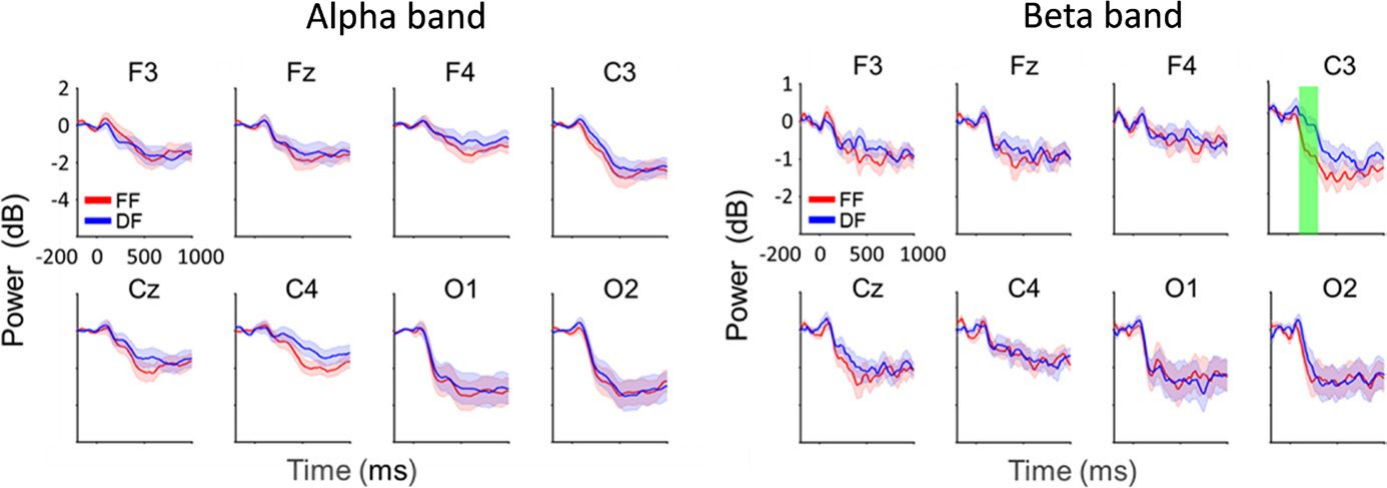
Time course of oscillatory brain activity in the alpha and beta bands during observation of favorite foods (FF) and disliked foods (DF). Significant differences in ERDs between FF and DF were observed in the beta band at 140–322 ms on C3 (shaded green area, *p* < 0.05 corrected by FDR)

Behavioral results showed no significant difference in response time between FF and DF trials (Figure 2). Oscillation analysis showed strong attenuation of ERD in the alpha and beta bands for both FF and DF trials (Figure 3). Thus, we further analyzed the ERDs in the alpha and beta bands. Figure 4 shows the time course of oscillatory brain activity in the alpha and beta bands for FF and DF trials. Significant differences in ERDs between FF and DF trials were observed in the beta band on C3 at 140–322 ms after the presentation of food images (shaded green area, *p* < 0.05 corrected by FDR). No significant difference was found in alpha band ERDs. Detailed statistical information regarding each time point of t value and effect size is shown in Figures S1 and S2.

**Figure 5:**
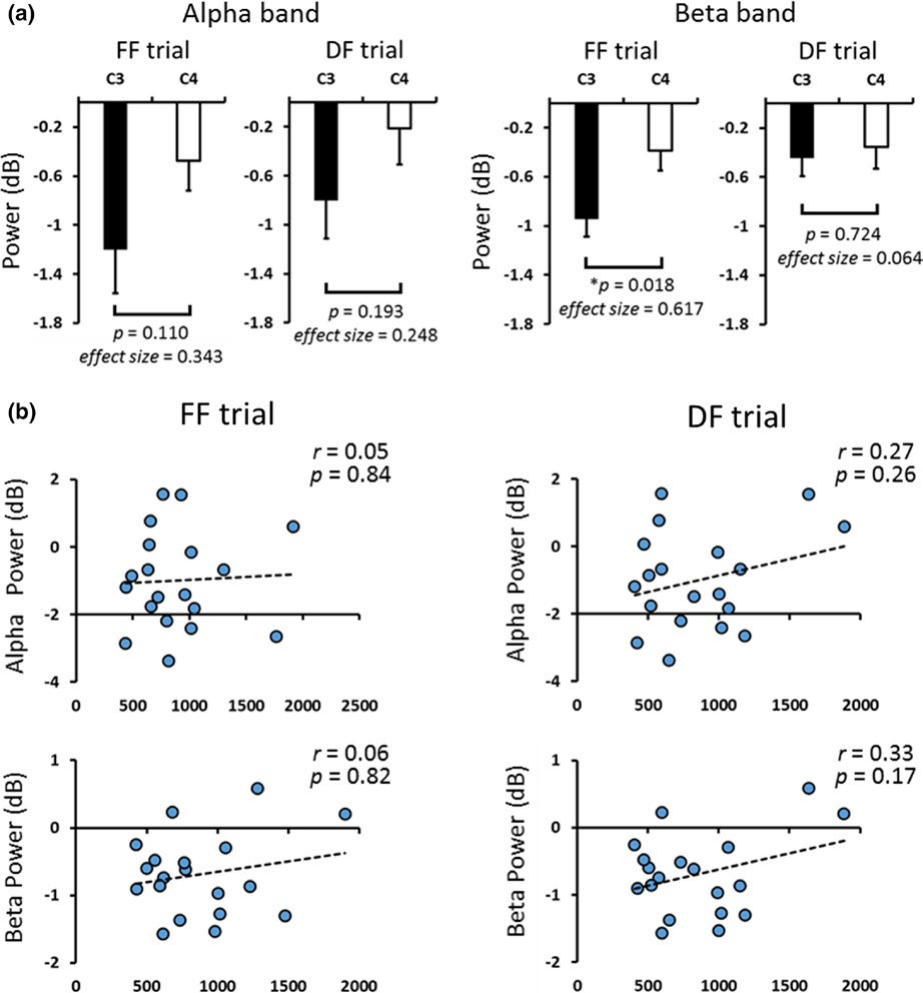
(a) ERDs on C3 and C4 areas at 0–500 ms after the presentation of food stimulus. In FF trial, ERDs in the beta band on C3 were significantly stronger than those on C4 (Student’s *t *test, *p* = 0.018). (b) Scatter diagrams showing ERDs on C3 areas at 0–500 ms and response time during FF and DF trials. There were no relationships between ERDs and response time (Pearson's correlation test, alpha: FF, *p* = 0.84, DF, *p* = 0.26; beta: FF, *p* = 0.82, DF, *p* = 0.17)

We examined the asymmetries of oscillatory brain activity on C3 and C4 channels during the observation of food stimulus. The results showed that ERDs in the beta band on C3 were significantly stronger than those on C4 (*p* < 0.05) in FF trial, whereas no such asymmetry was observed in the other frequency or condition (Figure 5a). In addition, correlation analysis between oscillatory brain activity on C3 area and response time showed no relationship in alpha and beta bands in FF and DF trials (alpha: FF, *p* = 0.84, DF, *p* = 0.26; beta: FF, *p* = 0.82, DF, *p* = 0.17; Figure 5b).

## 4 DISCUSSION

To clarify the effect of individual food preferences on oscillatory brain activity, we investigated the relationship between food preferences and oscillatory brain activity during the observation of food images. Our results showed significant differences in ERDs between FF and DF trials only in the beta band on C3 at 140–322 ms after the presentation of food images.

Several previous studies have reported a relationship between beta band oscillatory brain activity and preparation for swallowing (Dziewas et al., 2003; Jestrović et al., 2015; Maeda et al., 2004; Yoshikawa, Tanaka, Ishii, Fujimoto, & Watanabe, 2014; Yoshikawa et al., 2016). For instance, Yoshikawa et al. (2014) reported that the preparation stage of swallowing is characterized by ERDs in beta bands in the sensorimotor cortex. Dziewas et al. (2003) demonstrated strong left hemispheric dominance for activation of the primary sensorimotor cortex during volitional swallowing, whereas the swallowing reflex was less strongly lateralized to left hemispheric dominance. In addition, the somatosensory cortex is reported to play an important role in the execution of swallowing as well as the integration of sensorimotor information related to preparation for swallowing (Dziewas et al., 2003). The premotor cortex is also an important area for preparation of movement, including preparation for swallowing (Malandraki, Sutton, Perlman, Karampinos, & Conway, 2009; Martin et al., 2004; Sörös, Inamoto, & Martin, 2009).

In this study, a significant difference in ERDs between FF and DF trials was observed in the beta band over the left sensorimotor area. Previous studies have reported that ERDs reflect activated cortical areas and occur within a range of motor and sensory paradigms as well as cognitive paradigms (Neuper et al.,^2006^ ; Rektor et al., 2006; Klostermann et al., 2007; Dujardin, Bourrie, & Guieu, 1995). In addition, a study has shown that the localizations of ERDs recorded by electrophysiological methods and those of hemodynamic responses recorded by fMRI are concordant, supporting the notion that ERDs represent increased neural activation in the cortical area (Singh, Barnes, Hillebrand, Forde, & Williams, 2002). Furthermore, ERDs in the beta band of the sensorimotor area are known to reflect movement preparation (Rektor, Sochůrková, & Bocková, 2006; Klostermann et al., 2007). In this study, the significant ERD in the beta band peaked at 186 ms in FF trials. This result is consistent with a previous study using magnetoencephalography which found that ERDs in the beta oscillation related to swallowing changed significantly around 200 ms (Yoshikawa et al., 2014). In this study, participants were asked to rate their subjective level of preference for the food by pressing a button in the response phase. As mentioned above, ERDs in the alpha and beta bands occur not only during movement but also during the preparation of movement (Cheyne, 2013; Pfurtscheller & Aranibar, 1999; Pfurtscheller & Lopes da Silva, 1979). Thus, it was possible that ERDs observed in this study are attributed to the preparation of finger movement. To rule out this possibility, we analyzed response time between FF and DF trials. The result showed no significant difference in response time between the two trials. In addition, correlation analysis showed no significant relationship between ERDs in the oscillatory brain activity and response time, suggesting that ERDs observed in this study are attributed to visual food stimulus but not preparation of finger movement. Furthermore, ERDs in the beta band were significantly stronger for FF than DF, suggesting that oscillatory brain activity can be modulated by individual food preferences.

As described above, premotor and somatosensory area activity is closely related to movement preparation (Malandraki et al., 2009; Martin et al., 2004; Sörös et al., 2009; Sugata et al., 2017). In this study, there were no significant differences in ERDs between FF and DF trials on other EEG channels, except for C3 channel. Because frontal and occipital areas are associated with attentional and visual processing (Goodale, 2011; Stuss, 2006), respectively, we could rule out the possibility of different activation of attentional and visual processing between the observation of favorite and disliked foods. In addition, left hemispheric ERDs in the beta band during the observation of favorite food were significantly stronger than the right one. Considering that strong left hemispheric activation of the primary sensorimotor cortex is involved in volitional swallowing (Dziewas et al., 2003), our results may indicate that observing the favorite food more facilitates volitional swallowing than that of dislike food. Because the anticipatory stage of swallowing includes preparation for swallowing, the significant ERDs observed in the beta band on C3 in this study may reflect oscillatory brain activity related to preparation for swallowing in movement‐related cortical areas, including the M1, premotor area, and somatosensory area. Although we could not isolate the generator of ERDs among these regions due to low spatial resolution, individual food preferences may impact oscillatory brain activity related to preparation for swallowing during the anticipatory stage of swallowing. We hope that these results may lead to the development of new swallowing rehabilitation methods for patients with dysphagia that apply food preferences to modulate oscillatory brain activity.

However, this study has several limitations. First, this study did not address other frequency bands, such as delta, theta, and gamma bands. Because we wanted to examine the relationship between food preferences and oscillatory brain activity during the anticipatory stage of swallowing, we focused on the alpha and beta bands. However, recent studies have suggested the relationship of other frequency components with oscillatory brain activity, indicating that these frequencies contain important information related to movement and somatosensory functions (Cavanagh & Frank, 2014; Dalal et al., 2011; Dziewas et al., 2003; Yoshikawa et al., 2014). Therefore, neural oscillations of these frequencies may provide more detailed neural profiles related to the anticipatory stage of swallowing. Second, our results showed significant differences in beta ERDs between FF and DF on C3, and we believe that these differences may be due the effect of individual food preferences on movement preparation related to swallowing in premotor and sensorimotor areas. However, defining the strict mapping of motor and cognitive functions by oscillatory neural activity may be difficult because a variety of functions are derived from cortical oscillatory rhythms (Jestrović et al., 2015; Malandraki et al., 2009; Martin et al., 2004). Third, although we believed that individual food preferences commonly generate similar oscillatory patterns, we cannot rule out the possibility that these preferences generate individual brain responses because complex stimuli, in contrast to simple stimuli, such as checkerboards or beeps, can be perceived by the participants in a very different manner that reflects their personality. Thus, strictly defining the effect of food preferences on brain oscillations may be difficult. Thus, to reveal the detailed oscillatory neural profiles related to the relationship between individual food preferences and oscillatory brain activity during the anticipatory stage of swallowing, further study is needed.

## 5 CONCLUSION

This study revealed the relationship between individual food preferences and oscillatory brain activity during the observation of food images. Visual stimuli of favorite foods significantly modulated ERDs in the beta band relative to disliked food stimuli. This finding indicates that individual food preferences may impact oscillatory brain activity related to swallowing preparation during the anticipatory stage of swallowing.

## CONFLICT OF INTEREST

None of the authors declare any conflict of interest.
