# Supplementary material for: Effect of individual food preferences on oscillatory brain activity
Source: Brain Behav. 2019 Apr 4;9(5):e01262. doi: 10.1002/brb3.1262 (PMC6520299; doi:10.1002/brb3.1262)
Supplement: Supplementary file 1 [file BRB3-9-e01262-s001.pdf]

Supplementary Information for

Effect of individual food preferences on oscillatory brain activity

Nachie Tashiro[a], Hisato Sugata[b], Takashi Ikeda[c], Kojiro Matsushita[d], Masayuki Hara[e], Kenji Kawakami[b], Minoru Fujiki[a], Keisuke Kawakami[b]

[a]Department of Neurosurgery, Graduate School of Medicine, Oita University, Oita, Japan
[b]Faculty of Welfare and Health Science, Oita University, Oita, Japan
[c]Research Center for Child Mental Development, Kanazawa University, Kanazawa, Japan
[d]Department of Mechanical Engineering, Gifu University, Gifu, Japan
[e]Graduate School of Science and Engineering, Saitama University, Saitama, Japan

Contents:

Supplementary figures 1-2

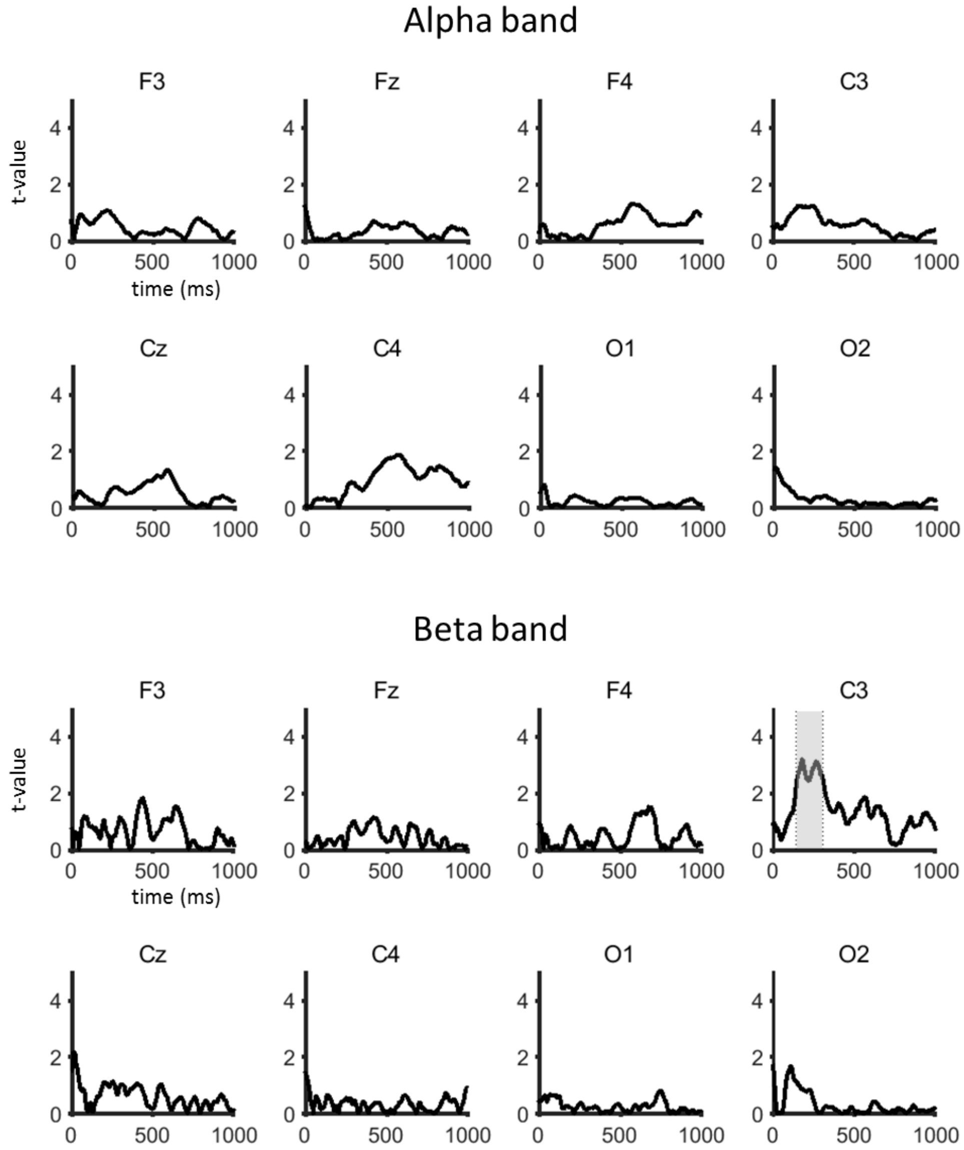

Figure S1. T values at each time point in the alpha and beta bands during the observation of favorite foods (FF) and disliked foods (DF). 0 ms corresponds to onset of food visual stimulus. Gray shaded area indicates time points of significant differences between FF and DF ($p < 0.05$).

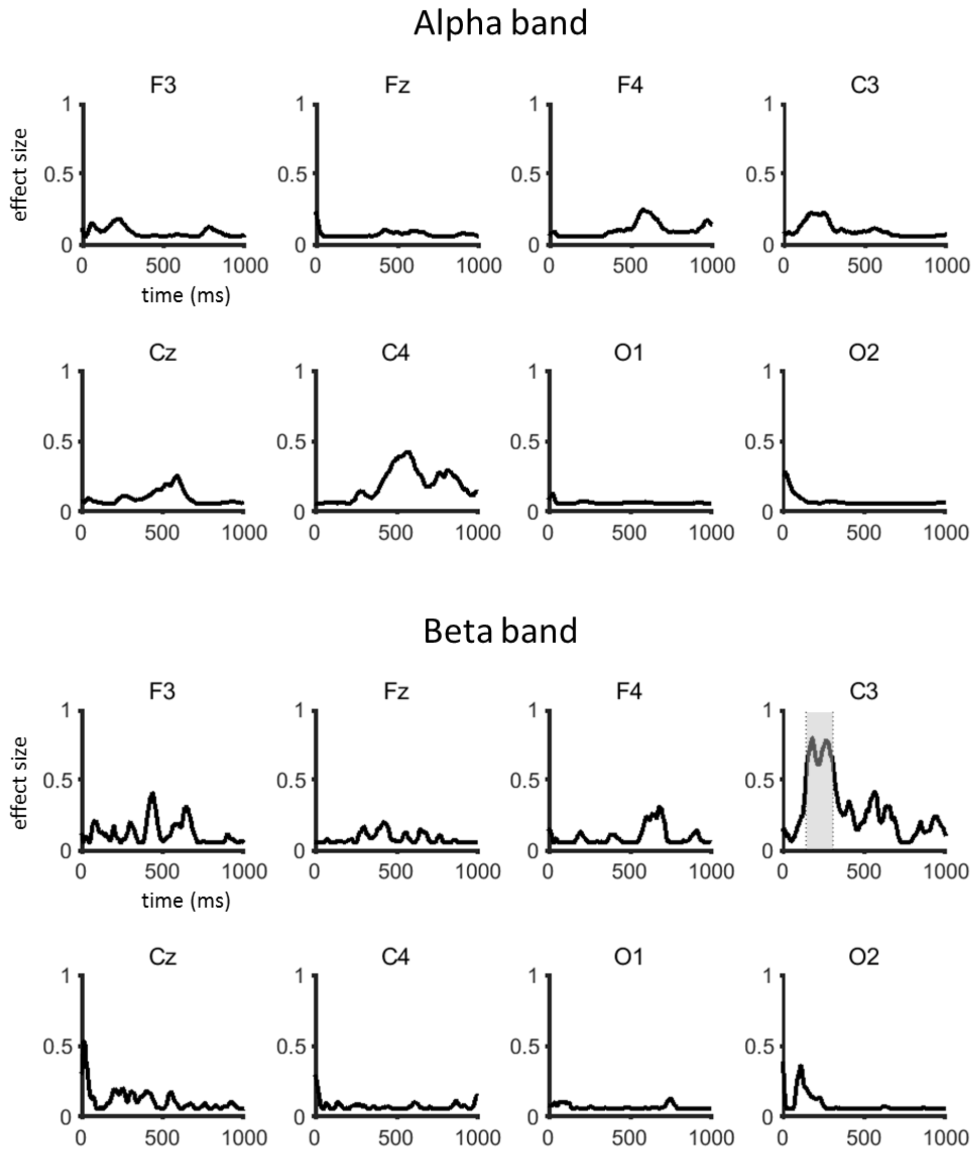

Figure S2. Effect size in each time point in the alpha and beta bands during the observation of favorite foods (FF) and disliked foods (DF). 0 ms corresponds to onset of food visual stimulus. Gray shaded area indicates time points of significant differences between FF and DF ($p < 0.05$).
